# Derivation of the Cramér-Rao Bound in the GNSS-Reflectometry Context for Static, Ground-Based Receivers in Scenarios with Coherent Reflection

**DOI:** 10.3390/s16122063

**Published:** 2016-12-05

**Authors:** Miguel Angel Ribot, Cyril Botteron, Pierre-André Farine

**Affiliations:** Electronics and Signal Processing Laboratory (ESPLAB), École Polythecnique Fédérale de Lausanne (EPFL), Maladière 71B (Microcity), Neuchâtel CH-2002, Switzerland; cyril.botteron@epfl.ch (C.B.); pierre-andre.farine@epfl.ch (P.-A.F.)

**Keywords:** GNSS-R, Cramér–Rao bound, CRB, coherent reflection, L-band, soil moisture estimation, altimetry, interference pattern technique, IPT

## Abstract

The use of the reflected Global Navigation Satellite Systems’ (GNSS) signals in Earth observation applications, referred to as GNSS reflectometry (GNSS-R), has been already studied for more than two decades. However, the estimation precision that can be achieved by GNSS-R sensors in some particular scenarios is still not fully understood yet. In an effort to partially fill this gap, in this paper, we compute the Cramér–Rao bound (CRB) for the specific case of static ground-based GNSS-R receivers and scenarios where the coherent component of the reflected signal is dominant. We compute the CRB for GNSS signals with different modulations, GPS L1 C/A and GPS L5 I/Q, which use binary phase-shift keying, and Galileo E1 B/C and E5, using the binary offset carrier. The CRB for these signals is evaluated as a function of the receiver bandwidth and different scenario parameters, such as the height of the receiver or the properties of the reflection surface. The CRB computation presented considers observation times of up to several tens of seconds, in which the satellite elevation angle observed changes significantly. Finally, the results obtained show the theoretical benefit of using modern GNSS signals with GNSS-R techniques using long observation times, such as the interference pattern technique.

## 1. Introduction

During the past two decades, researchers have been studying the use of the reflected Global Positioning System (GPS) and other Global Navigation Satellite Systems’ (GNSS) radio signals for remote sensing purposes [1]. This is commonly referred to as GNSS reflectometry (GNSS-R). The GNSS signals are transmitted at the L-band frequencies, which can penetrate cloud cover and are particularly sensitive to soil moisture, snow water content and sea-ice salinity. The GNSS-R concept was first proposed by Martin-Neira in [2], in 1993, for its application on ocean altimetry from airborne and spaceborne platforms. Since then, the use of GNSS-R for ocean altimetry, wind speed and sea state determination has gained considerable traction. Several works have been published describing specific GNSS-R instruments and architectures [3,4,5], retrieval techniques and algorithms [1,6], theoretical signal models [7,8,9,10], simulators [11,12,13] and several measurement campaigns carried out from different platforms [1,14,15]. Even specific GNSS-R space-based missions have been planned by different space agencies [3,16,17]. Furthermore, more remote sensing applications have been also proposed and validated through experimental campaigns, such as: soil moisture monitoring [7,18,19,20,21,22,23,24,25], snow monitoring [26,27,28,29,30,31,32], sea-ice monitoring [33,34], ground and calm water altimetry [35,36,37,38,39,40,41,42,43] and biomass estimation [44,45].

Why has GNSS-R gained this much attention? When compared to traditional radars used in remote sensing applications, which are typically monostatic and that make use of the backscattered signal, the GNSS-R receivers represent a non-cooperative passive bistatic (or multi-static) radar opportunity that makes use of the forward-scattered signals. Being a passive system, only the receiving part is required within a GNSS-R instrument, which implies lower cost, complexity and power consumption requirements than traditional active radars. Moreover, the high number of GNSS satellites, which is only expected to grow in the near future, enables global coverage from a network of transmitters whose location is very accurately known at any time instant. For air-borne and space-borne applications, the multi-static configuration allows for simultaneous surface sensing tracks, sensing more surface area within the same time and lower revisit times. However, the GNSS-R has some significant drawbacks when compared to traditional radars, mainly due to the GNSS signals’ features, i.e., their low power and relatively low bandwidth. Furthermore, we have no control over the transmitted signal features (e.g., modulation or code length).

The theoretical estimation precision that can be achieved using the different GNSS-R techniques is still under study. Early work on the statistical properties of the scattered GNSS signals and their connection to ocean altimetry and wind speed retrievals was described in [46,47]. This work adapted the classical approach described by Fischer et al. in [48] used to characterize the precision of radar altimeters and scatterometers. Later on, a similar approach was used again in [3], in an attempt to predict the precision of the PARISocean altimeter. Another attempt was put forward in [49], where the estimated delay difference error was computed using the approach described in [50], commonly used to characterize the delay error in traditional GPS with an early-prompt-late correlator tracking scheme. It is in [51] where the use of the Cramér–Rao bound (CRB) was firstly proposed in the GNSS-R ocean altimetry context. In [52], the authors computed the CRB using real measurements to estimate the scattered signal statistical properties, while later in [53,54], the authors used the CRB in an attempt to quantify the impact of the signal’s incoherent integration and the receiver’s front-end bandwidth, respectively, over the achievable precision.

Aside form specific GNSS-R instruments, multiple research works have demonstrated that in some scenarios, it is possible to process the data provided by static ground-based GNSS receivers, such as the ones used for geodetic applications, to directly perform GNSS-R. Under the assumption of a strong enough specular reflection, such that the phase coherence is partially preserved, it is possible to observe the interference between the line-of-sight (LOS) signal and the reflected signal. This interference can provide information about the geometry of the scenario and the properties of the reflection surface. A GNSS-R technique that exploits this information is the interference pattern technique or GNSS multipath reflectometry (GNSS-MR) (The two terms are arguably interchangeable. Nonetheless, some authors differentiate them by associating GNSS-MR with the cases where most of the interfering signals are co-polar (right-hand circularly polarized (RHCP)), while they use IPT for dedicated experiments, with dedicated experiments and sometimes a different polarization basis [1]). Either received by the same antenna, or combined later, if the two signal components have a delay difference shorter than a single modulation’s chip period, an interference pattern on the amplitude of the composite signal will be observed with the change of the GNSS satellite elevation with respect to the receiver. This interference pattern is mirrored into the receiver’s signal-to-noise ratio (SNR) estimate for the receiver channel tracking that signal [7,55,56]. By applying the IPT to the SNR data provided by the already deployed GNSS receivers, belonging to geodetic networks, it is possible to scout the surface surrounding the sites where these receivers are installed in a very cost-effective manner. Several snow and soil moisture monitoring experiments have been conducted using these data and the IPT, as well as some calm water altimetry experiments. However, to the best of our knowledge, little work has been published trying to characterize the estimation precision of these GNSS-R scenarios/techniques, where long observation times are considered [57,58]. The works previously mentioned, together with others such as [59,60], have focused on GNSS (mostly GPS) ocean scattered signals, which are mostly incoherent, i.e., with significant speckle noise.

In an attempt to fill this existing gap, in this paper, we make use of the CRB to provide the theoretical estimation bound for scenarios dominated by coherent reflection when the LOS and the reflected signals are received by the same antenna, both over short and long observation periods and for different GNSS signals: GPS L1 C/A and L5 and Galileo E1 and E5. In our previous work [57,58], the CRB was computed considering the samples at the output of the receiver’s prompt correlator during the tracking of the composite signal (i.e., LOS and the reflected component) as our measurements. In this paper, we assume no specific architecture for the GNSS-R receiver. The CRB is derived for the signal model corresponding to the samples obtained at the output of the receiver front-end. This implies that we are now considering all of the information contained in the received signal when computing the CRB, while in previous works, some information could be lost due to the processing of the received signal. Thus, the CRB expressions derived in this paper represent a more fundamental performance bound, independent of the GNSS receiver architecture considered.

This paper is organized as follows. Section 2 describes the GNSS-R scenario under consideration, summarizing all of the relevant assumptions made and later introducing the corresponding signal model for this scenario. Section 3 contains the derivation of the CRB for the LOS and an arbitrary number of multipath components over short measurement intervals, which is further adapted to our specific scenario using the Fisher information matrix (FIM) transformation approach. In Section 4, we analyze the impact of different parameters, e.g., the propagation path difference, over the CRB obtained. Section 5 extends the derivation of the CRB for long observation times, while in Section 6, we discuss the results obtained when computing this extended CRB as a function of the scenario parameters. Finally, Section 7 summarizes the results and highlights the paper’s main conclusions.

## 2. Scenario Definition

In this section, we describe the scenario for GNSS-R coherent reflections that we have considered for this work. By doing so, we summarize all of the relevant assumptions that are needed to define the received GNSS signal model at the output of the receiver front-end.

As expected, the properties of the reflected signal when impinging the receiver antenna will strongly depend on the scenario geometry and on the reflecting surface itself [61]. It is precisely that dependency that allows us to retrieve useful information from the signal. In this paper, we study a scenario similar to the one defined in [7].

**Figure 1:**
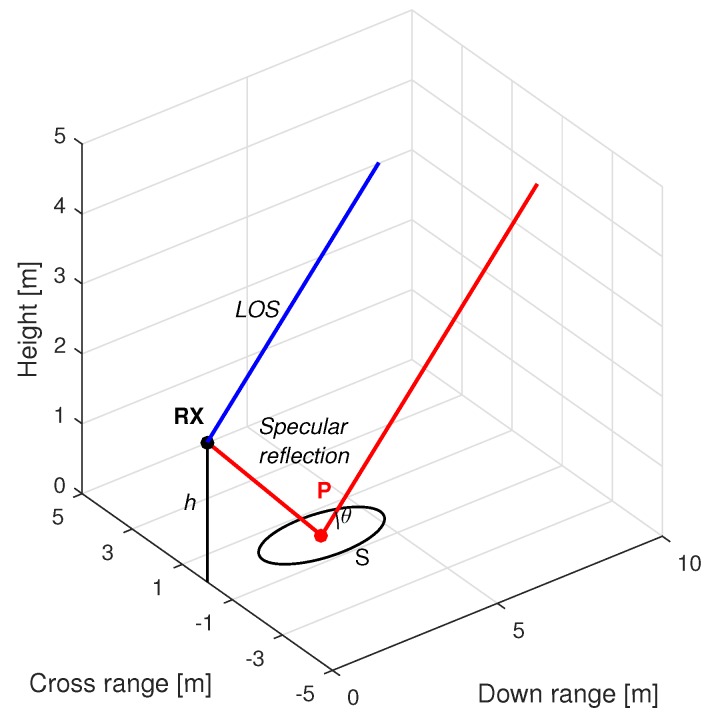
Scenario geometry depicting the LOS (**blue**) and the specular reflection (**red**) signal propagation paths. RX represents the position of the receiver’s antenna phase center, located at a height *h*, defined as its orthogonal distance to the reflection surface. The point *P* marks the specular reflection point. The values $\theta_{el}=30^{\circ }$ and h=2 m are used in this case as the example.

In such a scenario, the GNSS-R receiver is ground-based and stationary during the data acquisition. This receiver has a single antenna simultaneously receiving the line-of-sight (LOS) signal and a single reflected signal, where the latter has been forwardly scattered from the specular reflection point on the surface. The geometry of our scenario is shown in Figure 1. The reflection surface is assumed to be a horizontal planar surface with a homogeneous layered structure, i.e., the dielectric permittivity of the medium will only depend on the depth, i.e., the *z*-axis. These conditions could be fulfilled by surfaces, such as bare soil, snow- or vegetation-covered soil, or a calm water surface. Because of the large distance separating the satellite and the receiver, the elevation angle of the incident signal at the specular reflection point and at the receiver antenna can be considered both equal to the angle $\theta_{el}$. In addition, we consider the reflection surface in our scenario to be smooth according to the Rayleigh criterion [61], and thus, the standard deviation of the surface height $\sigma_{s_{h}}$, i.e., the surface roughness, satisfies:

(1) $$\sigma_{s_{h}}<\frac{\lambda }{8\sin\left(\theta_{el}\right)},$$

where *λ* is the carrier wavelength. By default, we will assume a $\sigma_{sh}=0.01$ m, low enough to fulfill the Inequality (1) for the GNSS L1 and L5 carrier frequencies and $\theta_{el}\leq 35^{\circ }$. The reflected signals are usually characterized as the sum of two contributions or components: one specular and another one diffuse [61]. Under the smooth surface assumption, the coherent component reflected from the specular reflection point will clearly dominate, and as a consequence, the contribution of the diffuse component will be disregarded in our analysis. Under such an assumption, we can consider the energy reflected mainly coming from the first Fresnel zone [7,61], and the reflected signal can be well approximated by a single delayed and complex-attenuated replica of the LOS signal. The first Fresnel zone describes an ellipse on the reflection surface (denoted as *S* in Figure 1) with:

(2) $$a=\frac{b}{\sin(\theta_{el})};\;b=\sqrt{\frac{\lambda \;h}{\sin(\theta_{el})}+\left(\frac{\lambda }{2\sin(\theta_{el})}\right)},$$

where *a* represents the semi-major axis, oriented towards the satellite’s azimuth; and *b* is the ellipse’s semi-minor axis. As was also pointed out in [7,61], the waves carrying the GNSS signals will acquire some degree of depolarization upon their reflection on dielectric mediums. While the wave corresponding to the LOS signal will be considered as purely right-hand circularly polarized (RHCP), the reflected wave can be described as a mix of both RHCP and left-hand circular polarization (LHCP) components.

**Figure 2:**
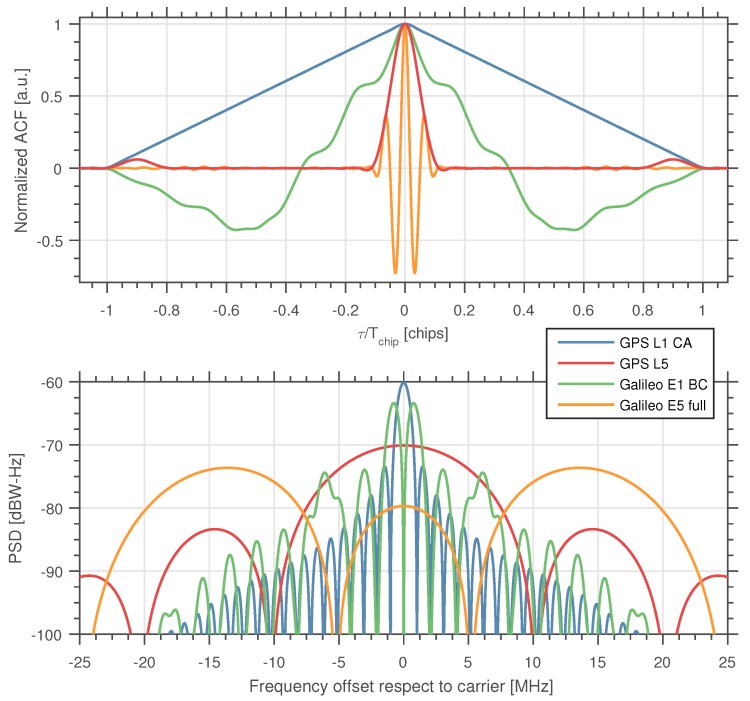
(**Top**) Normalized autocorrelation function of the transmitted GNSS signals considered (real part). The *x*-axis showing the delay *τ* has been normalized to $T_{chip}={\left(1.023\times 10^{6}\right)}^{-1}$ s; (**Bottom**) PSD of the signals. See Table 1 for more details.

The receiver’s antenna radiation pattern is assumed to be known, and its effect under different polarizations of the incoming signals is included within the modeling of the amplitude of the received signals. Furthermore, we make no assumption about the maximum height for the receiver’s antenna phase center position in our scenario, as long as the smooth criterion defined by Inequality (1) is still respected. Without loss of generality, we constrain our analysis to the single-satellite and single-signal scenario in each case. This assumption is reasonable given the low crosstalk among different GNSS signals, as well as the low cross-correlation among the different satellite spreading codes [62,63]. In this paper, we have focused only on the GPS L1 C/A and GPS L5 (both L5I and L5Q) signals, as well as on the Galileo E1 (E1B: data; and E1C: pilot) and the full Galileo E5 signals. Table 1 summarizes the characteristics of the signals considered, and Figure 2 shows their normalized autocorrelation (real part).

Although the GNSS signals in Table 1 have different typical received powers [64,65,66], for comparison purposes, we assume a default carrier-to-noise ratio (C/N_0_) of 45 dB-Hz for the LOS signal in all of the cases, before considering the antenna gain.

### Signal Model at the Output of the Receiver Front-End

For the single-satellite scenario described, we collect $x\in C^{N\times 1}$, a vector representing the complex baseband signal received, sampled at the output of the receiver’s front-end over a total observation time interval of $T_{obs}$ seconds. We assume the sampling rate $f_{s}$, such that $T_{obs}=Nf_{s}^{-1}$. The *n*-th sampling instant is defined as $t_{n}=t_{0}+nf_{s}^{-1}$, where $t_{0}$ is the time where the first measurement was taken. The vector x can then be expressed as:

(3) $$x=\underset{r}{\underset{︸}{\sum_{m=0}^{M-1}r_{m}}}+n,$$

where the GNSS signal, described by the vector r, has been expressed as *M* superimposed independent signal components $r_{m}$, each one corresponding to a different propagation path. In Equation (3), n is circularly-symmetric complex Gaussian noise with covariance C. Thus, x is a random vector distributed as $\mathrm{CN}\left(r,C\right)$. Each $r_{m}$ component can be expressed as:

(4) $$r_{m}=u_{m}d_{m}e^{j\phi_{m}},$$

where $u_{m}$ and $\phi_{m}$ are, respectively, the amplitude and the phase at the instant $t_{0}$ (referenced to the receiver’s local oscillator) of the *m*-th individual component. $u_{m}$ and $\phi_{m}$ are assumed to remain constant during observation intervals of up to a 1-s duration. These parameters already include the effects of the receiver antenna’s gain for each signal component. In Equation (4), the vector has been defined as:

(5) $$d_{m}≜{\left[d_{m}\left(t_{0}\right),\ldots ,d_{m}\left(t_{N-1}\right)\right]}^{T}\in C^{N\times 1},$$

with each of its component being equal to:

(6) $$d_{m}\left(t_{n}\right)=s\left(\alpha_{m}t_{n}-\tau_{m}\right)e^{j\omega_{c}\left(\alpha_{m}-1\right)t_{n}}.$$

$s\left(t\right)\in C$ represents the received filtered baseband navigation GNSS signal spread by the pseudorandom code of the satellite considered. In addition, for each component, we have that: $\tau_{m}$ is the time-delay at the instant $t_{0}$; $\alpha_{m}=\left(1+v_{m}/c\right)$ is the coefficient capturing the effect of the Doppler shift, where *c* is the signal propagation speed and $v_{m}$ the equivalent relative velocity between the transmitter and the receiver; and $\omega_{c}=2\pi f_{c}$, where $f_{c}$ is the signal carrier frequency. $\tau_{m}$ and $\alpha_{m}$ will also be assumed constant over time intervals of up to 1 s. This assumption for $\alpha_{m}$ is justified by the very low rate of change of the Doppler shift observed by a static receiver [67]. Given the maximum rate of change of the relative speed possible in this case, i.e., $dv_{m}/{dt|}_{Max}\approx 0.178$ m/s^2^ [67], $\alpha_{m}$ can vary as little as $\approx 5.94\times 10^{-10}$ within a 1-s interval.

In this paper, the front-end’s low-pass filter is implemented as a fifth-order Butterworth filter. The distortion introduced by this filter is already taken into account in the signal model s(t). The sampling frequency of the front-end is considered equal to the Nyquist rate with complex sampling, i.e., $f_{s}=2B_{fe}$, where $B_{fe}$ is the one-sided bandwidth (3 dB) of the low-pass filter. Finally, no quantization loss is considered in our analysis.

## 3. Derivation of the CRB for Short Observation Intervals

In this section, we derive the CRB given the output samples of the receiver’s front-end, whose signal model was defined in the previous section, over a total observation time $T_{obs}$ of up to 1 s. After introducing the CRB, we derive the general solution for *M* propagation paths. Then, we particularize the solution obtained for our scenario, described in Section 2, i.e., with M=2, corresponding to the LOS signal plus a single coherent specular reflection.

### 3.1. Definition and General Case for M Propagation Paths

Estimation of the error bounds plays a major role in parameter estimation in general, since they provide benchmarks to assess the performance of practical estimators. The workhorse in this respect is the well-known Cramér–Rao bound. The CRB defines a lower bound on the variance of any unbiased estimator for a set of deterministic parameters when the probability density function (pdf) of the measurements is known ([68], Chapter 3). The CRB quantifies the dependency of the measurements’ pdf on the unknown parameters to estimate. The stronger the pdf’s dependency on these parameters, the lower the CRB. The CRB for a vector of *L* unknown real-valued parameters $\xi \in R^{L\times 1}$ states that the covariance matrix of the estimates, $C_{\hat{\xi }}$, must satisfy (under standard regularity conditions):

(7) $$C_{\hat{\xi }}\geq J^{-1}\left(\xi \right),$$

where $J\left(\xi \right)$ is the Fisher information matrix (FIM), whose inverse is the CRB matrix. The elements in the FIM are defined as:

(8) $${\left[J\left(\xi \right)\right]}_{ij}=-E\left\{\frac{\partial^{2}\ln\;p\left(x;\xi \right)}{\partial \xi_{i}\partial \xi_{j}}\right\},$$

where lnp(x;ξ) is the log-likelihood function of ***ξ***, given the vector of random measurements x. Taking the inverse from Equation (8), we can express the CRB of the *i*-th parameter in ***ξ*** as:

(9) $$\mathrm{CRB}\left(\xi_{i}\right)={\left[J^{-1}\left(\xi \right)\right]}_{ii}.$$

In Section 2, we have introduced the vector x, modeling the discrete output of the receiver front-end during the time interval $T_{obs}$. As described there previously, the vector x is assumed to obey a circularly-symmetric complex Gaussian distribution. Its expected value, defined as ***μ***, will then be $r=\sum r_{m}$, as defined in Equation (3). We can express each signal component $r_{m}$ as a function of the following unknown parameters, grouped into the vector $\xi_{m}$ as:

(10) $$\xi_{m}≜{\left[u_{m},\phi_{m},\tau_{m},\alpha_{m}\right]}^{T},$$

where, for the *m*-th signal component, $u_{m}$ represents its amplitude, $\phi_{m}$ its phase shift, $\tau_{m}$ its time-delay or code phase and $\alpha_{m}$ its Doppler shift coefficient. In this case, we want to jointly estimate the vector $\xi \in R^{4M\times 1}$, which has been defined as the vector concatenating all of the $\xi_{m}$ vectors. Now, given the Gaussian distribution of the vector x assumed in our case, it is possible to make use of the Slepian–Bangs formula ([68], Chapter 3), which provides a convenient way to compute Equation (8) as:

(11) $${\left[J\left(\xi \right)\right]}_{ij}=\mathrm{tr}\left\{C^{-1}\frac{\partial C}{\partial \xi_{i}}C^{-1}\frac{\partial C}{\partial \xi_{j}}\right\}+2\text{ℜ}\left\{\frac{\partial \mu^{H}}{\partial \xi_{i}}C^{-1}\frac{\partial \mu }{\partial \xi_{j}}\right\},$$

where ℜ{·} denotes the real part of a complex vector or scalar, tr{·} denotes the trace operator, the superscript ^*H*^ is used to denote the conjugate transpose operator and the C represents the covariance matrix of x. Given that the noise component is independent from any of the parameters in ***ξ***, then the first term of the sum in Equation (11) will be equal to zero, due to $\partial C/\partial \xi_{i}=0$. In order to compute the remaining term in Equation (11), we can express the elements of the gradient of ***μ*** as:

(12) $$\begin{array}{cc} \frac{\partial \mu }{\partial u_{m}} & =d_{m}e^{j\phi_{m}}, \end{array}$$

(13) $$\begin{array}{cc} \frac{\partial \mu }{\partial \phi_{m}} & =ju_{m}d_{m}e^{j\phi_{m}}, \end{array}$$

(14) $$\begin{array}{cc} \frac{\partial \mu }{\partial \tau_{m}} & =u_{m}\frac{\partial d_{m}}{\partial \tau_{m}}e^{j\phi_{m}}, \end{array}$$

(15) $$\begin{array}{cc} \frac{\partial \mu }{\partial \alpha_{m}} & =u_{m}\frac{\partial d_{m}}{\partial \alpha_{m}}e^{j\phi_{m}}, \end{array}$$

where the vector $d_{m}$ is defined as in Equation (5). It is worth noticing that J(ξ) can be conveniently expressed as the following symmetric block matrix:

(16) $$J\left(\xi \right)=\left[\begin{array}{ccc} J_{\xi_{0}\xi_{0}} & J_{\xi_{0}\xi_{1}} & \ldots \\ J_{\xi_{0}\xi_{1}}^{T} & \ddots & \\ \vdots & & J_{\xi_{M-1}\xi_{M-1}} \end{array}\right],$$

where every element of each sub-matrix $J_{\xi_{m}\xi_{l}}$ is computed as:

(17) $${\left[J_{\xi_{m}\xi_{l}}\right]}_{ij}=2\text{ℜ}\left\{\frac{\partial \left(r_{m}^{H}\left(\xi_{m}\right)\right)}{\partial \xi_{i}}C^{-1}\frac{\partial \left(r_{l}\left(\xi_{l}\right)\right)}{\partial \xi_{j}}\right\},$$

with $\xi_{i}\in \left\{\xi_{m}\right\}$ and $\xi_{j}\in \left\{\xi_{l}\right\}$. Equation (17) can be used to compute the elements of J(ξ), and the CRB of ***ξ*** is straightforwardly obtained as in Equation (9). This case corresponds to the CRB under the assumption of Gaussian noise, independent of the received GNSS signal.

Let us now examine the case where only thermal noise is present. Since we have considered the sampling rate to be a multiple of the Nyquist sampling frequency, the samples in x can be considered uncorrelated, which implies a diagonal covariance matrix, i.e., $C=\sigma_{w}^{2}I$. The noise variance $\sigma_{w}$ can be computed as:

(18) $$\sigma_{w}^{2}=\frac{N_{0}}{2}\int_{-\infty }^{\infty }{\left|H_{Rx}\left(f\right)\right|}^{2}df=N_{0}B_{n}$$

where $N_{0}/2$ is the assumed white noise spectral density in W/Hz, $H_{Rx}\left(f\right)$ is the front-end’s normalized baseband filter response in the frequency domain and $B_{n}$ is defined as the equivalent noise bandwidth (one-sided). Thus, for complex white Gaussian noise (CWGN), we can express Equation (17) as:

(19) $${\left[J_{\xi_{m}\xi_{l}}\right]}_{ij}=\frac{2}{\sigma_{w}^{2}}\text{ℜ}\left\{\frac{\partial \left(r_{m}^{H}\left(\xi_{m}\right)\right)}{\partial \xi_{i}}\frac{\partial \left(r_{l}\left(\xi_{l}\right)\right)}{\partial \xi_{j}}\right\}.$$

If the narrowband approximation for the received signal is taken, i.e., $\alpha_{\left\{m,l\right\}}\approx 1$, then the CRB obtained as a result of inverting the matrix defined in Equation (16) is analogous to the results described in [69] (Chapter 4), where the CRB was derived for two propagation paths using a similar approach. In addition, Skournetou et al. used in [70] an approach similar to the one described in this paper, but for the case of up to four signal propagation paths.

### 3.2. Two Propagation Path Case for a Ground-Based Static Receiver

For the scenario described in Section 2, we assume M=2, with the $r_{0}$ component used to represent the line-of-sight (LOS) signal and $r_{1}$ used for the specular reflection. In this scenario, we consider a stationary receiver, where subsequently, the LOS and the specular reflection will experience the same Doppler shift; thus, $\alpha_{m}=\alpha_{l}:=\alpha$. This simplifies the FIM J(ξ) defined in Equation (16). Its elements related to $\alpha_{\left[m,l\right]}$ and a different parameter in ***ξ***, as defined in Equation (19), will now become zero. This implies that the estimates of *α* will be independent from the estimates of the rest of parameters, i.e., they will decouple. The same conclusion was reached in [69] (Chapter 4) when computing the CRB for two propagation paths. Having decoupled parameters allows us to disregard the estimation of the ***α*** for the rest of our analysis. The reason is that we are only interested in estimating the geometry of the scenario and the characteristics of the reflecting surface. In a stationary environment, ***α*** cannot be related with any useful information about the scenario that we want to retrieve. Thus, we are now redefining the vector of unknown parameters ***ξ***, by redefining each $\xi_{m}$ to just ${\left[u_{m},\phi_{m},\tau_{m}\right]}^{T}$.

For the scenario described in Section 2, we have only two propagation paths; using Equation (16), the FIM can be expressed as the 6×6 block matrix:

(20) $$J\left(\xi \right)=\left[\begin{array}{cc} J_{\xi_{0}\xi_{0}} & J_{\xi_{0}\xi_{1}}\\ J_{\xi_{0}\xi_{1}}^{T} & J_{\xi_{1}\xi_{1}} \end{array}\right],$$

where each submatrix, computed using Expression (19), can be expressed as:

(21) $$J_{\xi_{m}\xi_{l}}=\frac{2}{\sigma_{w}^{2}}\text{ℜ}\left\{\left[\begin{array}{ccc} d_{m}^{H}d_{l} & ju_{l}d_{m}^{H}d_{l} & u_{l}d_{m}^{H}\frac{\partial d_{l}}{\partial \tau_{l}}\\ -ju_{m}d_{m}^{H}d_{l} & u_{m}u_{l}d_{m}^{H}d_{l} & -ju_{m}u_{l}d_{m}^{H}\frac{\partial d_{l}}{\partial \tau_{l}}\\ u_{m}\frac{\partial d_{m}^{H}}{\partial \tau_{m}}d_{l} & ju_{m}u_{l}\frac{\partial d_{m}^{H}}{\partial \tau_{m}}d_{l} & u_{m}u_{l}\frac{\partial d_{m}^{H}}{\partial \tau_{m}}\frac{\partial d_{l}}{\partial \tau_{l}} \end{array}\right]e^{j\Delta \phi }\right\},$$

with m,l∈{0,1} and $\Delta \phi ≜\phi_{l}-\phi_{m}$.

Let us now introduce the discrete autocorrelation function (ACF) of s(t), defined as:

(22) $$R\left(\tau \right)≜\frac{1}{N}\sum_{n=0}^{N-1}s^{*}\left(\alpha t_{n}\right)s\left(\alpha t_{n}-\tau \right).$$

The superscript ^*^ is used to denote the complex conjugate.

Now, we introduce the following identities:

(23) $$\begin{array}{cc} R\left(\Delta \tau \right) & =\frac{1}{N}d_{m}^{H}d_{l}, \end{array}$$

(24) $$\begin{array}{cc} R^{\prime }\left(\Delta \tau \right) & =-\frac{1}{N}\frac{\partial d_{m}^{H}}{\partial \tau_{m}}d_{l}=\frac{1}{N}d_{m}^{H}\frac{\partial d_{l}}{\partial \tau_{l}}, \end{array}$$

(25) $$\begin{array}{cc} R^{\prime \prime }\left(\Delta \tau \right) & =-\frac{1}{N}\frac{\partial d_{m}^{H}}{\partial \tau_{m}}\frac{\partial d_{l}}{\partial \tau_{l}}. \end{array}$$

Notice that in order to keep our notation as simple as possible, we use $R^{\left(n\right)}\left(\Delta \tau \right)$ to indicate the *n*-th order partial derivative of *R* with respect to *τ* evaluated at the delay difference $\Delta \tau ≜\tau_{l}-\tau_{m}$. The Identities (23)–(25) are obtained straightforwardly by applying the chain rule, and they allow us to express Equation (21) as:

(26) $$J_{\xi_{m}\xi_{l}}=\frac{2N}{\sigma_{w}^{2}}\left[\begin{array}{ccc} R\left(\Delta \tau \right)\cos\left(\Delta \phi \right) & u_{l}R\left(\Delta \tau \right)\sin\left(\Delta \phi \right) & -u_{l}R^{\prime }\left(\Delta \tau \right)\cos\left(\Delta \phi \right)\\ -u_{m}R\left(\Delta \tau \right)\sin\left(\Delta \phi \right) & u_{m}u_{l}R\left(\Delta \tau \right)\cos\left(\Delta \phi \right) & u_{m}u_{l}R^{\prime }\left(\Delta \tau \right)\sin\left(\Delta \phi \right)\\ u_{m}R^{\prime }\left(\Delta \tau \right)\cos\left(\Delta \phi \right) & u_{m}u_{l}R^{\prime }\left(\Delta \tau \right)\sin\left(\Delta \phi \right) & -u_{m}u_{l}R^{\prime \prime }\left(\Delta \tau \right)\cos\left(\Delta \phi \right) \end{array}\right].$$

Now, since we can express $J_{\xi_{m}\xi_{l}}$ as in Equation (26), therefore J(ξ) will be a function of the ACF of s(t) and first and second order derivatives with *τ* evaluated at Δτ. It is helpful to notice that ACF and its derivatives can be expressed using their relationship with the signal’s power spectral density (PSD). By making use of the well-known Wiener–Khinchin theorem [68] (pp. 576–577) and the properties of the Fourier transform, we have that:

(27) $$R^{\left(n\right)}\left(\Delta \tau \right)=\int_{-\infty }^{\infty }{\left(j2\pi f\right)}^{n}S_{s}\left(f\right)e^{j2\pi f\Delta \tau }df,$$

where $S_{s}\left(f\right)$ represents the signal s(αt)’s PSD. Notice that s(αt) represents the filtered signal at the output of the receiver’s front-end, scaled in the time domain due to the Doppler effect. It already takes into account any distortion effect on the signal due to the front-end’s filtering. Thus, in our derivation, these effects are taken into account within the R() and its derivatives. Nonetheless, if we are interested in evaluating the influence of the front-end’s filtering on the resulting CRB, it might be more convenient to express $S_{s}\left(f\right)$ as:

(28) $$S_{s}\left(f\right)={\left|H_{Rx}\left(f\right)\right|}^{2}S\left(f\right),$$

where S(f) is the PSD of the signal received at the antenna. In the context of GNSS-R, in [54], the authors followed this approach to study the effects of using different front-end bandwidths and filter types in the estimation performance of the interferometric GNSS-R technique. Moreover, they considered also the band limitations of the signal transmitted in practice. In contrast, in our approach, we generate a model of s(t) simulating the samples at the output of the front-end, and then, we compute its ACF and its derivatives.

The selected front-end bandwidth, $B_{fe}$, will define the sharpness of the normalized autocorrelation peaks, which will have a great impact on its derivatives, $R^{\left(n\right)}$. In addition, the total number of samples considered within $T_{obs}$ will also depend on $B_{fe}$, as $N=T_{obs}/\left(2B_{fe}\right)$. If we consider the support of R(Δτ) to be approximately limited to the interval $\left[-T_{c},T_{c}\right]$, where $T_{c}$ is the chip duration, then, according to Equation (26), we have $J_{\xi_{m}\xi_{l}}\approx 0$ for propagation path differences greater than $\rho_{chip}=T_{c}c$, where *c* is the signal propagation speed. This is a consequence of the autocorrelation properties of PRNcodes [62]. Moreover, $J\left(\xi \right)$ becomes a diagonal matrix, which implies that all of the parameters in ***ξ*** are now uncoupled. From the FIM calculation point of view, this is equivalent to the scenario of having a receiver with two synchronized RF front-end channels, with uncorrelated noise components, and two different antennas: an up-looking one, exclusively receiving the LOS signal, and a second down-looking antenna, exclusively receiving the reflected component.

### 3.3. Introducing Phase Coherence Using FIM’s Parameter Transformation

So far, we have derived an expression for the FIM of ***ξ*** given the scenario defined in Section 2. However, in the GNSS-R context, we are actually more interested in a different set of unknown parameters that can be related to the geometry of the scenario, as well as to the properties of the reflecting surface. Moreover, up to this point, we have not taken into account in our signal model the phase coherence presented by specular reflection. This implies that the LOS and the reflected signals maintain a fixed phase relationship with each other and with respect to the local oscillator clock, used as the reference, i.e., $\phi_{1}=g\left(\phi_{0}\right)$.

In our approach, we have decided to compute the CRB for ***ξ*** and then transform it for a new set of parameters **Ψ**. This transformation of J(ξ) allows us to conveniently introduce the phase coherence assumption. We define the new vector of unknown parameters as:

(29) $$\Psi ={\left[u_{0},\phi_{0},\rho_{0},\left|\Gamma \right|,\phi_{\Gamma },h\right]}^{T},$$

where $u_{0}$, $\phi_{0}$, $\rho_{0}$ are the amplitude, phase and propagation distance (ρ=τc) for the LOS signal component. $\Gamma =\left|\Gamma \right|e^{j\phi_{\Gamma }}$ is the reflected signal coefficient, i.e., the complex attenuation experienced by the reflected signal when compared to the LOS. Its module is defined as:

(30) $$\left|\Gamma \right|=\frac{u_{1}}{u_{0}}.$$

Γ takes into account the surface reflectivity coefficient, the antenna complex gain and the polarization mismatch. Thus, it will depend on the satellite elevation angle ($\theta_{el}$) and on the azimuth angle of arrival of the reflected signal to the antenna. Since $\theta_{el}$ varies very slowly [67], we will assume it to remain constant for observation periods up to 1 s. *h* is the height of the receiver, defined as the orthogonal distance between the antenna phase center and the reflection surface. In our flat surface scenario, this height *h* relates to the propagation path difference between the LOS and the reflected component Δρ as:

(31) $$h=\frac{\Delta \rho }{2\sin\left(\theta_{el}\right)},$$

where Δρ is defined as $\Delta \rho =\left(\tau_{1}-\tau_{0}\right)c$. In addition, because of the coherence assumption on the reflected component, the phase difference between the two components, i.e., $\Delta \phi =\phi_{1}-\phi_{0}$, can be expressed as:

(32) $$\Delta \phi =\phi_{\Gamma }-\frac{2\pi }{\lambda }\Delta \rho ,$$

where *λ* is the carrier wavelength.

Following the approach described in [71], we can express the FIM for **Ψ** as:

(33) $$J\left(\Psi \right)=GJ\left(\xi \right)G^{T},$$

where G is the Jacobian transformation matrix defined as:

(34) $$G=\left\{\frac{\partial \xi_{j}}{\partial \Psi_{i}}\right\}.$$

The expressions for each of the elements of G are provided in Appendix A. The CRB for the parameters in **Ψ** can be directly obtained as the diagonal elements of $J^{-1}\left(\Psi \right)$. This two-step approach, i.e., first computing the FIM for ***ξ*** and then transforming it, has been used for its simplicity. It allows us to redefine the bound for a different set of parameters and to introduce more assumptions into our signal model without having to derive the FIM expression from scratch.

## 4. Analysis of the CRB for Short Observation Intervals

The matrix $J^{-1}\left(\Psi \right)$ derived in the previous section shows that CRB(Ψ) in the specular reflection scenario considered will depend on:

i The scenario geometry: through the propagation path difference between the two signal components Δρ, which will be a function of *h* and the satellite elevation angle $\theta_{el}$ (and its variation) during the observation time.
ii The properties of the reflecting surface: through its reflection coefficient, Γ, that can be modeled as a function of the signal’s incident angle and the electrical properties of the surface and its roughness.
iii The GNSS signal considered and the receiver features: the signal modulation and its transmission bandwidth, the C/N_0_ of the LOS signal, the antenna’s radiation pattern and the receiver front-end’s bandwidth (and the filtering scheme considered) will all have an impact on the resulting CRB.

However, understanding these dependencies only by examining $J^{-1}\left(\Psi \right)$ is not straightforward. The expressions for their diagonal elements can be, in the general case, quite cumbersome with several terms. Nonetheless, we examine the specific case when $\Delta \rho >\rho_{chip}$, where the initial FIM J(ξ) is a diagonal matrix, which greatly simplifies the terms in the final $J^{-1}\left(\Psi \right)$.

### 4.1. CRB when $\Delta \rho >\rho_{chip}$

In this particular case, not only the diagonal terms for the resulting $J^{-1}\left(\Psi \right)$ matrix, i.e., ${\left[J^{-1}\left(\Psi \right)\right]}_{ii}$, are much simpler, but this assumption is particularly relevant because it represents the best estimation case, i.e., when its CRB lower bounds all other cases with shorter Δρ. As shown in Appendix A, for *h* and |Γ| in **Ψ**, after some simple algebra, we obtain the following CRB expressions:

(35) $$\begin{array}{cc} \mathrm{CRB}\left(h\right) & =-\frac{1}{8\gamma \;{\mathrm{SNR}}_{0}\;\sin^{2}\left(\theta_{el}\right)\left(\frac{R^{\prime \prime }\left(0\right)}{c^{2}}\right)}, \end{array}$$

(36) $$\begin{array}{cc} \mathrm{CRB}\left(|\Gamma |\right) & =\frac{{\left|\Gamma \right|}^{2}+1}{2\;{\mathrm{SNR}}_{0}\;R\left(0\right)}, \end{array}$$

where the coefficient *γ* introduced has been defined as:

(37) $$\gamma =\frac{{\left|\Gamma \right|}^{2}}{{\left|\Gamma \right|}^{2}+1},$$

and ${\mathrm{SNR}}_{0}$ represents the equivalent SNR as if only the LOS signal was present and, as such, can be expressed as:

(38) $${\mathrm{SNR}}_{0}=\frac{u_{0}^{2}}{\sigma_{w}^{2}}N=2\frac{C}{N_{0}}T_{obs}.$$

Notice that the amplitude $u_{0}$ takes into account the effects of the receiver’s antenna gain pattern for the received LOS signal, which is dependent on the signal incident angle, i.e., a function of $\theta_{el}$.

From examining Equations (35) and (36), we can directly deduce the following:

1. With little surprise, both CRB expressions are inversely proportional to SNR_0_. However, CRB(h) is also inversely proportional to $\sin^{2}\left(\theta_{el}\right)$. This poses a trade-off: we require low elevations for the smooth surface assumption to hold, but at the same time, the lower the elevation, the higher the CRB(h). Moreover, as shown by Equation (2), low elevation angles imply larger first Fresnel zone areas, which decrease the spatial resolution of our estimates.
2. The values that CRB(|Γ|) can take for different |Γ| values are bounded, as a consequence of having defined Γ as a ratio, with 0<|Γ|≤1.
3. In both CRB expressions, the effects of the assumed received signal bandwidth and the front-end filtering (possible losses and distortions) are modeled within the $R^{\prime \prime }\left(0\right)$ and the ACF’s peak, i.e., R(0). $R^{\prime \prime }\left(0\right)$ can be understood as the curvature or the sharpness of the R(0). The higher the front-end bandwidth, the sharper the resulting ACF peak and, thus, the higher $R^{\prime \prime }\left(0\right)$. This holds as long as the front-end bandwidth is narrower than the received signal bandwidth, otherwise there will be no sharpening on the resulting R(0). Additionally, for a higher front-end bandwidth, the ${\mathrm{SNR}}_{0}$ will also increase if the Nyquist sampling rate assumption is maintained, as a consequence of having more samples for the same $T_{obs}$.

**Figure 3:**
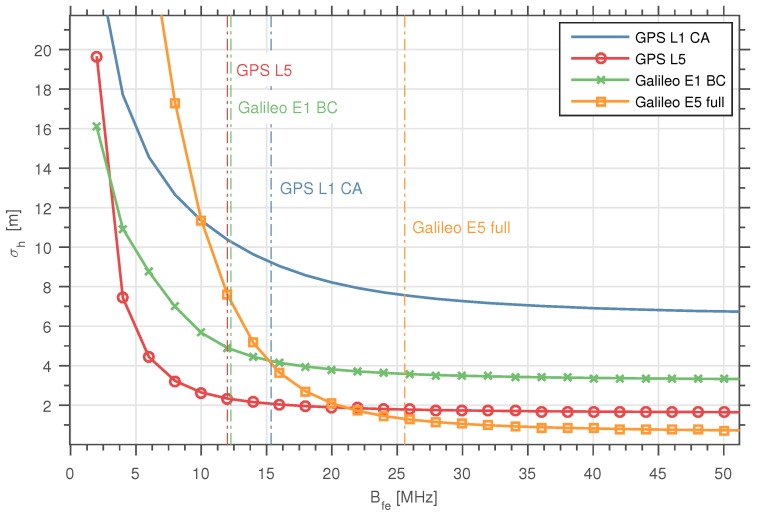
$\sqrt{\mathrm{CRB}(h)}$ as a function of the front-end’s bandwidth for the different GNSS signals considered. The vertical dashed lines are used to represent the $B_{Tx}$ (one-sided) considered for each of the signals.

In Figure 3, we show the results obtained when computing the CRB for *h* using Equation (35), as a function of the front-end’s bandwidth $B_{fe}$ for the different GNSS signals considered. Note that in Figure 3, the *y*-axis represents (the convention of representing the $\sqrt{\mathrm{CRB}}$ in the figures is followed consistently along the entire paper) $\sigma_{\Psi_{i}}=\sqrt{\mathrm{CRB}(\Psi_{i})}$. An isotropic antenna pattern was assumed, and |Γ| was set to $\sqrt{0.1}$, which corresponds to an attenuation of 10 dB in the reflected signal power with respect to the LOS. The $T_{obs}$ considered was equal to 1 s. The Δρ was fixed to 600 m to ensure isolation between the LOS and the reflected signal components provided by the code correlation properties. We observe how the CRB of *h* consistently decreases with the increase of $B_{fe}$ for all GNSS signals studied, as long as $B_{fe}$ remains lower than the (one-sided) signal bandwidth $B_{Tx}$. The slight decrease of the CRB of *h* for all GNSS signals observed in Figure 3 for $B_{fe}>B_{Tx}/2$ is explained due to the use of a non-ideal low-pass filter, like the one explained in Section 2, to model the limited transmission bandwidth. Also from Figure 3, we see how the $\sqrt{\mathrm{CRB}(h)}$ stays in the meter order, except for the Galileo E5 signal, which reaches decimeter order precision when $B_{fe}>40$ MHz, due to its highest bandwidth.

**Figure 4:**
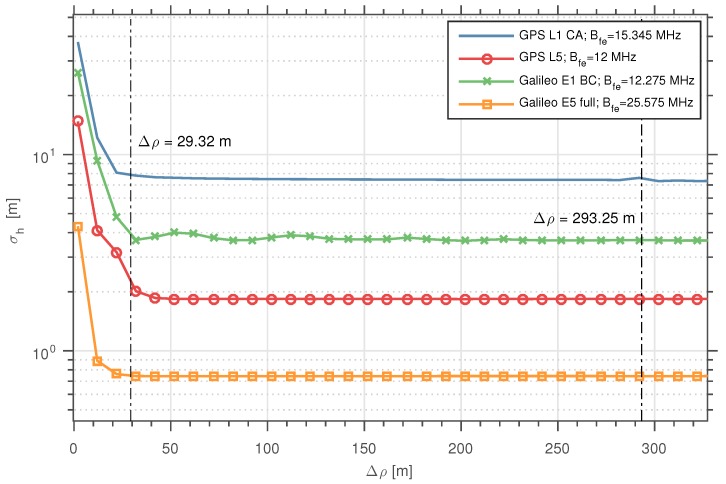
$\sqrt{\mathrm{CRB}(h)}$ as a function of Δρ for the different GNSS signals considered. $B_{fe}$ is the bandwidth of the receiver’s front-end assumed for each signal, set to match the signal bandwidth, i.e., $B_{Tx}/2$ (see Table 1). The two vertical lines represent the chip lengths: 293.25 m, of the GPS L1 CA and Galileo E1 BC signals; and 29.32 m, of the GPS L5 and Galileo E5 signals.

The CRB for the rest of parameters in **Ψ** is jointly obtained when computing $J^{-1}\left(\Psi \right)$. In Equation (32), we have expressed the phase difference between the two signal components as the sum of two contributions: $\phi_{\Gamma }$ and (2π/λ)Δρ, with the latter capturing the shift due to the extra propagation path corresponding to the specular reflection. For short observation intervals, where both of these contributions to Δϕ remain constant, it is not possible to individually estimate them precisely. This issue is easily spotted by examining the $\mathrm{CRB}\left(\phi_{\Gamma }\right)$ expression, i.e., the fifth element in ${\left[J^{-1}\left(\Psi \right)\right]}_{ii}$, derived in Appendix A, that can be approximated as:

(39) $$\begin{array}{cc} \mathrm{CRB}\left(\phi_{\Gamma }\right) & \approx -\frac{c^{2}k^{2}R\left(0\right)}{2\;\gamma \;{\mathrm{SNR}}_{0}\;R^{\prime \prime }\left(0\right)}, \end{array}$$

where k=2π/λ is the wavenumber for the received signal carrier. Since the numerator in Equation (39) will usually be many orders of magnitude higher than its denominator, resulting in $\sqrt{\mathrm{CRB}\left(\phi_{\Gamma }\right)}\gg 2\pi$ rad, this implies that we cannot estimate $\phi_{\Gamma }$ properly. Given this ambiguity in individually estimating the two contributions to Δϕ, performing precise phase altimetry is only possible if $\phi_{\Gamma }$ is assumed to be known or small enough that it can be neglected. Otherwise, a bias in the altimetry estimates is introduced. Thus, even if we considered the phase coherence in our derivation of CRB(*h*), Expression (35) is equivalent to the CRB for pure GNSS-R code altimetry only, i.e., as if no phase information were used. The CRB for code altimetry can be also derived following the same proposed approach, described in the previous section, but just assuming no phase coherence between the LOS and the reflected signal, i.e., considering Δϕ as the difference between two independent phases when computing the G transformation matrix defined in Equation (34), which leads to the same results as in Equation (35). In addition, in [72,73], in an effort made by the authors to characterize the code-tracking accuracy of GPS receivers, they computed the minimum code thermal noise jitter when using noncoherent delay-lock loop (DLL) discriminators, i.e., $\sigma_{\mathrm{DLL}}^{2}$. The expressions provided in their results are very frequently used to characterize the tracking performance of different receiver architectures [62] (Chapter 5). Additionally, we can use these same expressions to compute the minimum variance on the *h* estimate, i.e., $\sigma_{\hat{h}}^{2}$, in a straightforward way, as:

(40) $$\sigma_{\hat{h}}^{2}\geq \frac{c^{2}\left(\sigma_{\mathrm{DLL}-0}^{2}+\sigma_{\mathrm{DLL}-1}^{2}\right)}{4\sin^{2}\left(\theta_{el}\right)},$$

where $\sigma_{\mathrm{DLL}-0}^{2}$ and $\sigma_{\mathrm{DLL}-1}^{2}$ are the minimum variances of the LOS and the reflected signal code delays, respectively. In Equation (40), two independent tracking channels are assumed: one tracking the LOS signal and the other tracking the reflected signal. The results when computing Equation (40) match the CRB(*h*) values obtained using Equation (35) when $\Delta \rho >\rho_{chip}$, which correspond to the flat part of the curves later shown in Figure 4. As a consequence, it follows that a two-tracking channel architecture for code altimetry is efficient in terms of the bound defined in Equation (35) given a sufficiently large propagation path difference. In practice, such a scenario might appear to be unlikely for ground-based receivers. Nonetheless, many of the new GNSS signals have higher bandwidths, which implies lower $\rho_{chip}$ values (e.g., 293 m for GPS L1 C/A signal vs. 29.3 m for GPS L5) [62].

In the previous discussion, we have highlighted the problem arising when trying to separately estimate the two contributions to Δϕ and how the CRB for the joint estimation of **Ψ** captures this effect. However, what if we assume some prior knowledge of one of the two terms in Δϕ? We examine now the impact on the CRB if we assume that one of these terms is known, which represents the best case scenario given such an assumption. We study two cases: (1) when $\phi_{\Gamma }$ is assumed to be known, referred to as “phase altimetry” case; and (2) when *h* is known, referred to as “reflection coefficient estimation” case.

#### 4.1.1. Phase Altimetry

In this case, we assume the angle $\phi_{\Gamma }$ as known. Now, in order to obtain the new expression ${\mathrm{CRB}}_{alt}\left(h\right)$, we remove the row and the column of J(Ψ) associated with $\phi_{\Gamma }$ and then compute the matrix inversion $J^{-1}\left(\Psi \right)$, as is described in Appendix A. We obtain the following expression:

(41) $$\begin{array}{cc} {\mathrm{CRB}}_{alt}\left(h\right) & =\frac{1}{8\gamma \;{\mathrm{SNR}}_{0}\;\sin^{2}\left(\theta_{el}\right)\left(k^{2}R\left(0\right)-\frac{R^{\prime \prime }\left(0\right)}{c^{2}}\right)}\\ & \approx \frac{1}{32\lambda \;{\mathrm{SNR}}_{0}R\left(0\right)}{\left(\frac{\lambda }{\pi \sin\left(\theta_{el}\right)}\right)}^{2}, \end{array}$$

by making the approximation of $R^{\prime \prime }\left(0\right)/c^{2}$ very small (e.g., ≈0.1 for the worst case, i.e., the Galileo E5 signal with $B_{fe}=B_{Tx}$) compared to $k^{2}R\left(0\right)$ (e.g., ≈623), given the signal bandwidths considered. The phase information is now effectively used and the bound multiple orders of magnitude lower. As expected, it is directly proportional to $\lambda^{2}$, i.e., the lower the carrier wavelength, the higher the possible precision of the *h* estimate. In addition, we observe that when comparing Equation (41) with Equation (35), the dependence on $R^{\prime \prime }\left(0\right)$ can be neglected now. This implies that in this phase altimetry case, the estimation performance of the phase altimetry techniques should be negligibly impacted by the bandwidth of the signal used. As a consequence, the use of the new GNSS with higher bandwidths, but with the same carrier frequency, should bring little improvement to the achievable precision of the phase altimetry techniques for short $T_{obs}$ when $\Delta \rho >\rho_{chip}$.

#### 4.1.2. Reflection Coefficient Estimation

Analogously, we compute now ${\mathrm{CRB}}_{r}$ for |Γ| and $\phi_{\Gamma }$ (subindex *r*), but in this case, we proceed by removing the row and the column associated with *h*, as shown, once again, in Appendix A. We obtain the following expressions:

(42) $$\begin{array}{cc} {\mathrm{CRB}}_{r}\left(|\Gamma |\right) & =\mathrm{CRB}\left(|\Gamma |\right), \end{array}$$

(43) $$\begin{array}{cc} {\mathrm{CRB}}_{r}\left(\phi_{\Gamma }\right) & =\frac{1}{2\;\gamma \;{\mathrm{SNR}}_{0}\;R\left(0\right)}. \end{array}$$

As expected, the ${\mathrm{CRB}}_{r}\left(|\Gamma |\right)$ is the same as in the joint estimation of **Ψ**, while ${\mathrm{CRB}}_{r}\left(\phi_{\Gamma }\right)$ is now orders of magnitude smaller when compared to Equation (39) and well bellow 2π, thus implying that in this case, it is possible to obtain useful phase estimates.

### 4.2. CRB when $\Delta \rho <\rho_{chip}$: Interference Case

When $\Delta \rho <\rho_{chip}$, we cannot process anymore the two signal separately, which was otherwise possible thanks to the correlation properties of the PRN codes. Now, the initial FIM J(ξ) is no longer a diagonal matrix, and the analytic expressions for ${\left[J^{-1}\left(\Psi \right)\right]}_{ii}$ become quite cumbersome. Thus, from now on, we will rely on numerical evaluation of ${\left[J^{-1}\left(\Psi \right)\right]}_{ii}$ to compute the CRB.

In Figure 4, we show the results of computing CRB(h) as a function of the Δρ for the different GNSS signals under study. Once again, we have considered a ${\left|\Gamma \right|}^{2}=0.1$, and $T_{obs}=1$ s. As expected, we have observed that when Δρ increases, the bound reaches the values obtained using Equation (35). Moreover, we also observe how that the CRB reaches these values even for $\Delta \rho \ll \rho_{chip}$. In summary, we can identify two consequences of having higher bandwidth signals: (a) the asymptotic value for the bound is reached faster; and (b) the value of the bound is lower for any Δρ. In the case of (a), this can be clearly related with having a sharpened peak of the ACF for higher front-end bandwidths.

**Figure 5:**
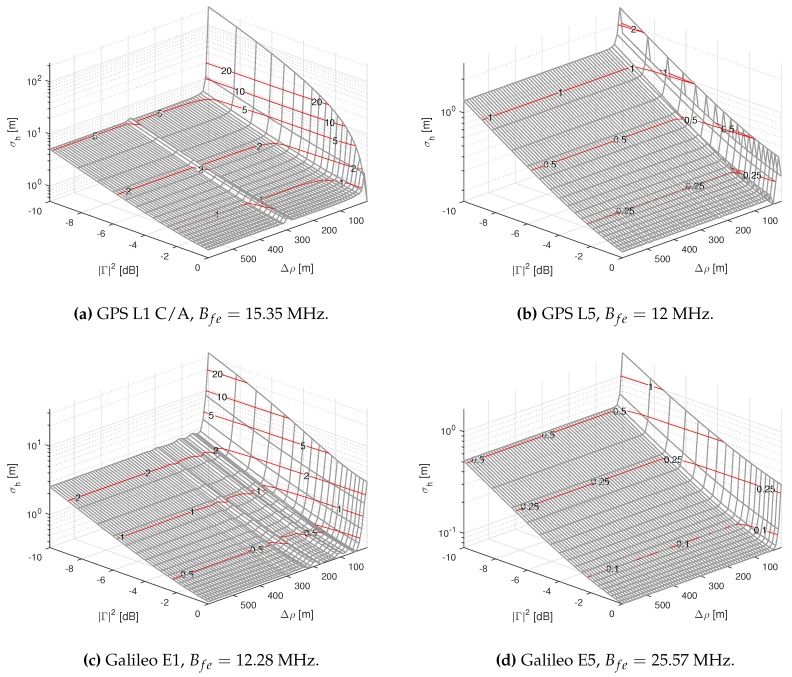
$\sqrt{\mathrm{CRB}(h)}$ for different ${\left|\Gamma \right|}^{2}$ and Δρ values for the considered GNSS signals. Red lines are used to represent the level curves, each one labeled with its corresponding value of $\sqrt{\mathrm{CRB}(h)}$ (in m). $B_{fe}$ represents the assumed front-end’s one-sided 3-dB bandwidth.

In Figure 5, we show the results of computing CRB(h) for different Δρ and |Γ| values. The plots presented allow us to visually determine, approximately, the order of the best achievable precision given a Δρ and |Γ| combination. The *y*-axis represents ${\left|\Gamma \right|}^{2}$, which in practice is equivalent to the gain experienced by the reflected signal compared to the LOS. As expected, for Δρ values higher than $\rho_{chip}$, the CRB agrees with the one obtained with Equation (35). Figure 4 confirms that the CRB when $\Delta \rho >\rho_{chip}$ lower bounds all of the other cases, as we hypothesized at the beginning of this section.

## 5. Derivation of the CRB for Long Observation Intervals: CRB($\Psi_{long}$)

So far, we have assumed total observation times of up to one second. However, the IPT uses much longer observation times, usually on the order of several minutes. Can we use the CRB expressions obtained in Section 3.3 and Section 4 for such long observation times? Unfortunately, we cannot use them for observation times $T_{obs}$ longer than one second. This is due to the fact that, for $T_{obs}>1$ s, the approximation described in Section 2, of considering the parameters in **Ψ** to be constant over $T_{obs}$, is no longer valid. In this case, the change in the delay of the received LOS signal cannot be well approximated anymore as a linear expression with an initial value of $\tau_{0}$ s. In addition, neither the Doppler shift observed, nor the phase shift $\phi_{0}$ with respect to the receiver oscillator can be considered as constant. That is due to the non-linear relative movement of the satellite and the receiver over long observation times and due to the signal propagating through the atmosphere, which effects the signal as it varies with time [62]. In addition, the direction in which the signals impinge on the antenna will vary over time as a consequence of the variation of the satellite’s elevation, i.e., $\theta_{el}$, and the azimuth angles observed. Subsequently, this variation can result in a change in the LOS signal amplitude $u_{0}$ and an extra phase shift, i.e., a variation of $\phi_{0}$, since these two parameters also model the effect the receiver’s antenna radiation pattern.

In realistic scenarios, modeling the reflection coefficient Γ, even for homogeneous and horizontal flat surfaces, can be a complex matter. Nonetheless, we still can characterize Γ as a function of the vector $s_{\Gamma }$, containing the set of parameters related to the reflection surface physical properties; the signal’s impinging angle on the surface; and the antenna gain towards the specular reflection point [61]. The two last terms can be expressed as a function of $\theta_{el}$. Thus, we have that:

(44) $$\Gamma \left(\theta_{el}\right)=f\left(\theta_{el};s_{\Gamma }\right).$$

If we consider *h* and the parameters in $s_{\Gamma }$ constant over $T_{obs}\gg 1$ s, then we can compute the CRB for a new vector of unknown parameters $\Psi_{long}$, defined as:

(45) $$\Psi_{long}≜{\left[u_{0}^{T},\phi_{0}^{T},\tau_{0}^{T},s_{\Gamma }^{T},h\right]}^{T},$$

where $u_{0}$, $\phi_{0}$ and $\tau_{0}$ are column vectors of *L* elements, which we refer to as “fast-varying” parameters. In our analysis, these parameters are associated with the values of $u_{0}$, $\phi_{0}$ and $\tau_{0}$ at the *l*-th time interval of $T_{coh}=$ 1 s duration, with $L=T_{obs}/T_{coh}$ being the total number of these intervals for $T_{obs}$. We assume that $\theta_{el}$ remains constant over each $T_{coh}$ interval. The previous assumption is based on the fact that the rate of change of $\theta_{el}$ over time observed by ground-based static receivers is very low, i.e. on the order of $10^{-3}{\;}^{\circ }/s$ [67].

We can now use the same FIM parameter transformation approach to compute the $\mathrm{CRB}(\Psi_{long})$, which was introduced in Section 3.3. This approach allows us to obtain $J(\Psi_{long})$ as a function of the J(Ψ) for each of the different *L* short time intervals $T_{coh}$. In order to do so, we first define the vector $\Psi_{ext}$ of unknown parameters as:

(46) $$\Psi_{ext}≜{\left[\Psi_{0}^{T},...,\Psi_{L-1}^{T}\right]}^{T},$$

where each subvector $\Psi_{l}$ corresponds to the *l*-th $T_{coh}$ observation interval. We build now the matrix $J\left(\Psi_{ext}\right)$ as:

(47) $$\begin{array}{cc} J\left(\Psi_{ext}\right) & ≜\left[\begin{array}{ccc} J\left(\Psi_{0}\right) & & 0\\ & \ddots & \\ 0 & & J\left(\Psi_{L-1}\right) \end{array}\right] \end{array}$$

(48) $$\begin{array}{cc} & =\mathrm{diag}\left\{J\left(\Psi_{0}\right),\ldots ,J\left(\Psi_{L-1}\right)\right\}, \end{array}$$

where each $J(\Psi_{l})$ is the FIM computed using Equations (20)–(34) for the *l*-th $T_{coh}$ observation interval. The $J\left(\Psi_{ext}\right)$ generated is equivalent to assuming all of the parameters in any $\Psi_{l}$ are independent from any of the parameters in a different subvector $\Psi_{k}$. This assumption is only used as a means to introduce the parameter dependencies between observation intervals by transforming $J\left(\Psi_{ext}\right)$, once again, using a new Jacobian transformation matrix $A\in R^{6L\times P}$, defined as:

(49) $$A=\left\{\frac{\partial \Psi_{ext_{j}}}{\partial \Psi_{long_{i}}}\right\},$$

where *i* and *j* are the indexes used for the rows and columns, respectively. The number of columns in the matrix A, *P*, is equal to the number of parameters in $\Psi_{long}$, i.e., 3L+1+ the number of elements in $s_{\Gamma }$. Since the parameter *h* was already in **Ψ** and is considered constant for all of the *L* intervals, $\partial h_{l}/\partial h=1$, for any *l*. Finally, $J(\Psi_{long})$ is obtained as:

(50) $$J\left(\Psi_{long}\right)=A\;J\left(\Psi_{ext}\right)A^{T}.$$

A more detailed description of how the matrix A is constructed is provided in Appendix A. The CRB can be obtained directly as ${\left[J^{-1}\left(\Psi_{long}\right)\right]}_{ii}$. Furthermore, we shall point out that the previous derivation is independent of the model of Γ considered, as long as the gradients of |Γ| and $\phi_{\Gamma }$ with respect to $s_{\Gamma }$ exist. No assumption was made regarding the number of parameters in $s_{\Gamma }$. Nonetheless, it is well known that the CRB can only increase with the number of unknown parameters considered, which intuitively makes sense, given the increased uncertainty.

## 6. Analysis of the CRB($\Psi_{long}$)

In this section, we compute the CRB, using Equation (50), for different simulation cases. Our goal is to study the effect of the received signal’s satellite elevation span covered during the observation time ($\Delta \theta_{el}$), as well as the impact of the GNSS signal modulation, over the theoretical achievable precision in the estimation of the parameters in $\Psi_{long}$.

The variation of $\theta_{el}$ during the observation time has the following implications:

1. The propagation path difference Δρ will change due to the displacement of the specular reflection point. In addition, the angle of arrival of both the LOS and the reflected component to the antenna will also vary.
2. If the reflection coefficient Γ is assumed to depend on $\theta_{el}$, this will also change during the observation time.

Up to this point, we have not selected any specific model for the reflection coefficient Γ. From now on, we will use the model for a single-layer bare soil introduced in [7], which we will call the Zavorotny–Larson model (Z-L). This model is described in more detail in Appendix B, and it allows us to specify Γ as a function of $\theta_{el}$ and the following parameters:

(51) $$s_{\Gamma }={\left[\varepsilon_{r},\sigma \right]}^{T},$$

where $\varepsilon_{r}$ and *σ* are the real part of the surface’s relative permittivity and the surface conductivity, respectively, which are assumed constant over the entire observation interval. Notice that this model for Γ is used as an example in our analysis and that the approach proposed to obtain $J(\Psi_{long})$ can accommodate other models for $\Gamma (\theta_{el};s_{\Gamma })$.

In addition, the gain of the receiving antenna will have an important impact over the CRB. To assess this impact, we consider two possible antenna cases in our analysis:

1. An ideal case with an isotropic antenna model.
2. A Leica GNSS AR25 antenna [74] model. Only the antenna gain’s amplitude has been modeled. The antenna phase center is assumed to be located at the distance *h* perpendicular to the reflection surface. The antenna boresight is pointing upwards, parallel to the surface normal vector (i.e., an elevation of 90°). We have used the pattern model provided as part of the open source GPS multipath simulator [75].

In both cases, we make the assumption that the antenna complex gain is accurately known and that all of the available signal’s energy is considered by defining the receiver’s front-end bandwidth as $B_{fe}=B_{Tx}/2$.

### 6.1. CRB($\Psi_{long}$) vs. $\Delta \theta_{el}$

**Figure 6:**
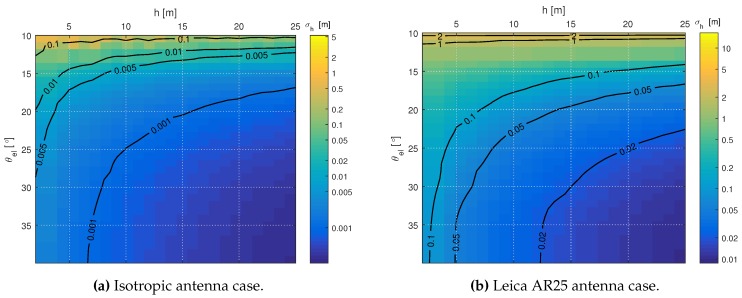
$\sqrt{\mathrm{CRB}(h)}$ for different $\Delta \theta_{el}$’s (vertical axis), starting from 10°, and true receiver heights *h* (horizontal axis). Complete left-hand circular polarization (LHCP) reflection with constant ${\left|\Gamma \right|}^{2}=-10$ dB is assumed. (**a**) The results when an ideal isotropic antenna is considered, while (**b**) shows the results when the Leica AR25 antenna pattern is considered. Black lines are used to represent different level curves for the values labeled on top of them.

In this first case, we compute the CRB for different *h* values and elevation spans $\Delta \theta_{el}$, covering from $\theta_{el}=10{\;}^{\circ }$–$35{\;}^{\circ }$. In the results obtained, the first individual $\Delta \theta_{el}$ value represents a special case, since it corresponds to a single short observation interval of $T_{obs}=1$ s, with no change in $\theta_{el}$. In this case, the bound matches the results obtained using the short-period CRB derived in Section 3. Figure 6 shows the CRB of *h* results for the isotropic antenna and the AR25 antenna cases, with the GPS L1 C/A signal. In the figure, the highest CRB values are located at the left-upper corner of the plot, which correspond to the lower values of antenna heights (below 5 m) and elevation spans (below 15°). The CRB values decrease almost monotonically with both the increase of *h* and the elevation span covered. As expected, it becomes clear from the plots that the antenna pattern has a very significant impact on the CRB of *h* given the same *h* and $\Delta \theta_{el}$. Figure 6a shows that sub-millimeter precision could be achieved in theory in the ideal isotropic antenna case. On the other hand, in the AR25 antenna case, the plot in Figure 6b shows that now, only centimeter precision can be achieved. These CRB values now obtained are significantly lower than the CRB values computed previously in Section 3. Firstly, given the satellite elevation rate of $5\times 10^{-4}{\;}^{\circ }/$s considered, the observation time is above the hour duration for $\Delta \theta_{el}>18^{\circ }$. The longer the observation time considered, the more independent samples we collect, thus the lower CRB values obtained. Secondly, the CRB computed now takes into account that the phase difference information can be used for the estimation of *h* in this case. Intuitively, this can be explained by the different rate of change of the two contributions to the phase of the reflected component: the phase shift due to the scenario geometry (related to *h*) and the phase shift due to the polarization change due to the reflecting surface properties, i.e., to Γ.

Having a higher true *h* has two main implications. First, the higher the *h*, the faster the oscillation rate of the interference pattern observed in the composite amplitude of the received signal with the change of satellite elevation. Second, the Δρ will be higher, which makes it easier to differentiate between the two signal components, given the code autocorrelation properties, and the resulting CRB can be lower, as we have shown in Section 3. However, techniques like the IPT only use the output of the estimated SNR or the prompt correlator (single output), and they can only work if there is interference between the LOS and the reflected signal, i.e., if $\Delta \rho <\rho_{chip}$ [21]. Thus, with IPT, we face a trade-off: we require low Δρ values in order to observe the interference pattern, but according to the CRB expressions obtained, low Δρ values degrade the achievable estimation precision.

### 6.2. Effects of the Signal Modulation on the CRB($\Psi_{long}$)

We now present the results of computing the CRB of $s_{\Gamma }$ and *h*, given $\Delta \theta_{el}$ spans of different lengths and the GNSS signals considered. For each signal, we have considered a particular $B_{fe}\;=\;B_{Tx}/2$. We have considered the Leica AR25 antenna model and a true receiver height h=3 m. We also have used the Z-L model for $\Gamma (\theta_{el})$ given a dry ground surface with a permittivity (real part) $\varepsilon_{r}=30$ and a conductivity σ=0.2 S/m [76]. The initial $\theta_{el}$ is $10^{\circ }$ for all of the elevation spans considered.

**Figure 7:**
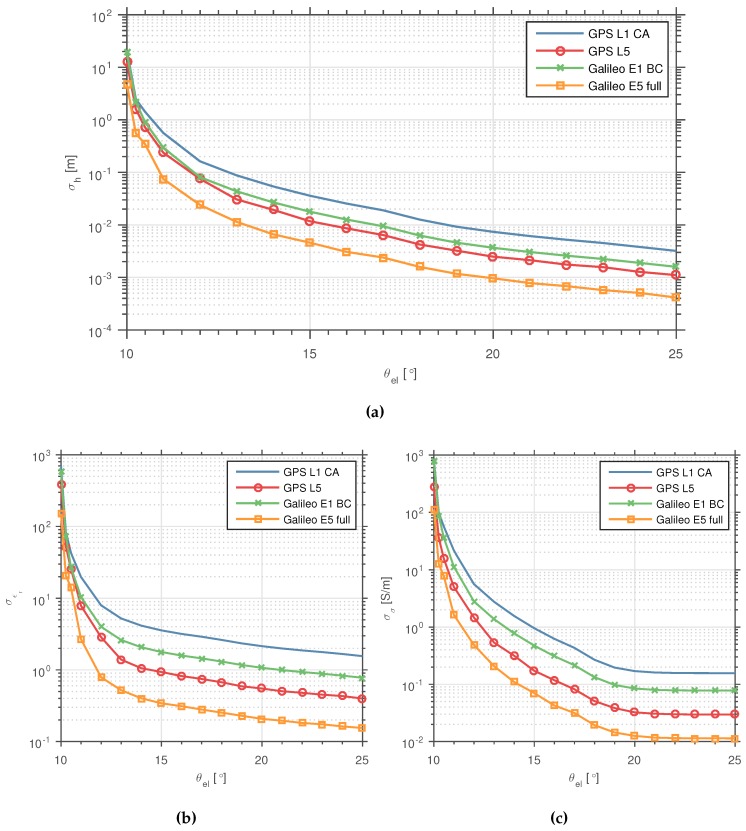
$\sqrt{\mathrm{CRB}}$ for (**a**) *h*, (**b**) $\varepsilon_{r}$, and (**c**) *σ*, for different $\Delta \theta_{el}$ starting from an initial $\theta_{el}=10^{\circ }$. Each data point in the plots represents the results for a $\Delta \theta_{el}$, with its value on the *x*-axis representing the final $\theta_{el}$ angle achieved in that span. The first data point (left of the plot) corresponds to a single $T_{coh}$ observation.

**Figure 8:**
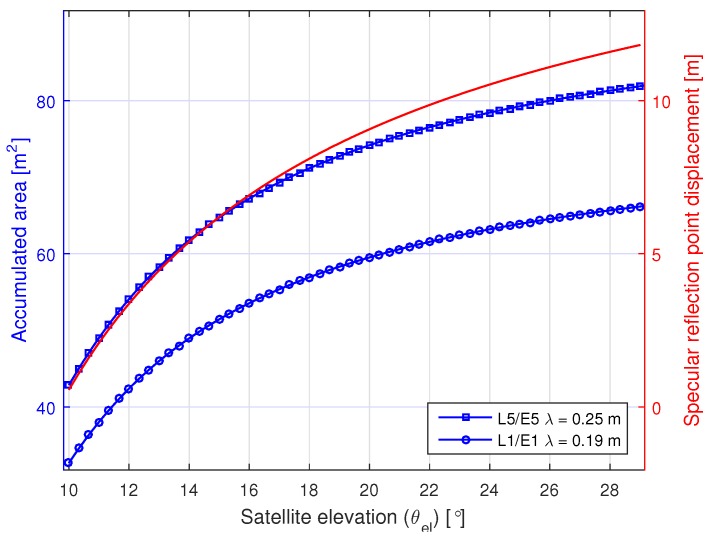
Accumulated area within the first Fresnel zone for h=3 m with the variation of $\theta_{el}$. The right axis shows the displacement of the specular reflection point from its initial location when $\theta_{el}=10^{\circ }$. The GPS L5 and Galileo E5 signals have slightly different carrier frequencies (1176.45 MHz and 1191.795 MHz, respectively), but both of their wavelengths have been approximated to the same λ=0.25 m.

The results obtained are summarized in Figure 7. As expected, the longer the $\Delta \theta_{el}$ covered during the observation time, the lower the CRB values obtained, for all three parameters. However, this improvement is not linear. We observe how the CRB decreases significantly for $\Delta \theta_{el}$ covering up to ∼2°, while it decreases at a much slower rate when extending $\Delta \theta_{el}$ beyond that value. This observed behavior can be explained as follows. For our scenario and given the GNSS signals’ carrier wavelength, i.e., from 0.19–0.25 m, a $\Delta \theta_{el}$ of ∼2° is required to observe a 2π phase shift in the reflected signal phase, caused exclusively due to the change in Δρ. In the IPT context, this corresponds to observing a single complete oscillation of the SNR interference pattern. Longer $\Delta \theta_{el}$, i.e., longer observation times still provide some improvement. It is important to remark about the existing trade-off between extending $\Delta \theta_{el}$, i.e., extending the observation time, and the displacement of the specular reflection point over the reflection surface. The change in $\theta_{el}$ also changes the size of the first Fresnel zone. For low elevation angles, such as the ones that we need to consider to ensure the smooth surface assumption, the change in the area covered by the first Fresnel zone and the displacement of the specular reflection point is significant, as shown in Figure 8. In practice, it is unlikely to find real surfaces that are that flat and homogeneous (and with no obstacles), except maybe for the case of calm water surfaces. On the other hand, the antennas used in practice (e.g., in geodetic sites) have lower cross-coupling at lower elevation angles, i.e., less attenuation on the LHCP signal for these elevations. This is why higher oscillation amplitudes of the SNR interference pattern are observed for $\theta_{el}$ angles in standard GNSS geodetic receivers [7,55].

We shall also notice that even for long observation times, where the signal’s phase information can be used in the parameter estimation, the greater the signal’s bandwidth, the lower the CRB for all of the parameters. This is due to the fact that for a h=3 m, Δρ is small enough for the two signal components to interfere, and the separability provided by the PRN code’s correlation properties acts only partially. This separability is needed to measure the code delay difference between the two components, which is required to unambiguously estimate *h*. This cannot be done by only using the phase difference information.

We would like to highlight that, if during the $\Delta \theta_{el}$ observed, the Γ coefficient changes significantly, e.g., when the Brewster angle is covered by $\Delta \theta_{el}$ [21,61], lower values of the CRB for $\varepsilon_{r}$ and/or *σ* can be achieved with shorter $\Delta \theta_{el}$. From the CRB point of view, if the pdf of the observed signal changes significantly with the variation of the unknown parameters, such as $\varepsilon_{r}$ and/or *σ*, then it is easier to estimate these parameters from the observed samples, resulting in lower CRB values. Likewise, when the Γ coefficient barely changes during the observation time considered, it becomes harder to unambiguously retrieve the $\varepsilon_{r}$ and/or *σ* parameters from the observed samples, thus resulting in higher CRB values.

Figure 7 shows that centimeter precision can be theoretically achieved when estimating *h* over elevation spans larger than 20° for all GNSS signals. The CRB also shows that millimeter precision can be theoretically achieved for the $\Delta \theta_{el}=\left[10^{\circ },25^{\circ }\right]$ in the case of the Galileo E5 signal, i.e., using its full bandwidth. In practice, however, it is unlikely to find real surfaces that are that flat and homogeneous (and with no obstacles), maybe except for very calm water surfaces. Thus, the millimeter precision is unlikely to be achieved by any estimator in a practical scenario, even with the assumptions made in this paper (e.g., perfect knowledge of the antenna radiation pattern, the satellite elevation angle, etc.). The asymptotic behavior observed over long observation times of the CRB of the conductivity *σ* can be related to the small dependency and change of Γ during the considered elevation spans for the dry ground surface.

## 7. Conclusions and Future Work

In this work, we have derived a theoretical precision bound for the GNSS-R coherent reflection scenario, for ground-based static receivers. The CRB has been derived first for observation times of up to 1 s (short observation intervals), and later on, we have extended this bound for long observation intervals of up to hundreds of seconds, where the satellite elevation angle was changing during the observation time. The CRB was computed for GPS and Galileo signals with different bandwidths and different modulations, i.e., BPSK, CBOC (6,1,1/11) and AltBOC (15,10). For short observation times and $\Delta \rho >\rho_{chip}$, we obtained simple analytic expressions of the CRB for the parameters of interest. Later on, the CRB was extended for long observation times by using the FIM transformation approach. The approach followed can easily accommodate different models for the surface’s reflection coefficient Γ. In this paper, we selected the Z-L model to describe Γ, and then, we have computed the CRB as a function of satellite elevation spans of different sizes covered during the observation time. The impact of the antenna radiation pattern was considered within the modeling of Γ. The specific case that we have studied (receiver height, antenna pattern and surface properties) is an example selected to understand the dependency of the CRB on the signal properties and the scenario parameters.

For short observation times of up to 1 s, the results obtained have shown that even for antennas designed to mitigate multipath and these antennas being installed with their boresight perpendicular to the ground, it is still theoretically possible to obtain meter precision (or even decimeter in the case of using the full Galileo E5) given a front-end with a large enough bandwidth. In the case of long observation intervals, the CRB computed confirms the link between the variation of the satellite elevation and the achievable precision. Moreover, the CRB evaluation presented confirms the cm level precision that has been reported by many works, e.g., [29,32,38,41,77,78,79], among others, some of them using standard GNSS geodetic stations [80]. Reasonable accuracy was obtained in the estimation (joint) of $\varepsilon_{r}$ and *σ* over observation times covering satellite elevation variations above 10°. According to the CRB results computed, the modernized GNSS signals considered in this work, i.e., GPS L5, Galileo E1 and Galileo E5, can potentially provide better estimation precision for all of the parameters when compared to the GPS L1.

In this work, the CRB results presented have been mainly focused on the *h* estimation. Using the approach described in this paper, further study of the CRB for the parameters related to the reflection surface’s properties is planned, in order to characterize the precision achievable in other GNSS-R applications, such as soil moisture determination. In addition, two main future activities are planned to continue with the work described in this paper: first, to further explore the CRB for similar scenarios with different models for the Γ and, second, to compute the CRB for the scenarios where experimental campaigns were carried out, as a way to fully validate the bound obtained and assess the performance of the techniques used.

As a final remark, we want to point out that a practical limitation of the precision bounds provided in this paper is the fact that our signal model (for coherent reflection) might not accurately match the true reflected signal. In many practical scenarios, the reflected signal will be better described as a combination of the coherent and incoherent components. The importance of each component will depend on the considered scenario. In any case, the CRB results provided in this paper will lower bound those obtained with a signal model considering also the presence of incoherent reflections. We are considering as future work the derivation of CRB expressions for different kinds of ground-based receiver scenarios, which is not straightforward from the analytic point of view.

## Figures and Tables

**Table 1:** GNSS signal characteristics considered [64,65,66]. $f_{c}$ is used to refer to the signal’s carrier frequency; $f_{chip}$ is the modulation’s primary code chip rate; and $B_{Tx}$ is the considered transmitted signal bandwidth. $B_{Tx}$ has been selected to be equal to the receiver’s reference bandwidth, defined in the corresponding Interface Control Documents for each signal. For the GPS L1 C/A signal, we have considered the reference bandwidth defined for the Block III satellite vehicles.

Signal	$f_{c}$ (MHz)	Modulation	$f_{\mathrm{chip}}$ (MHz)	Main Lobe BW(MHz)	$B_{\mathrm{Tx}}$ (MHz)
GPS L1 C/A	1575.42	BPSK (1)	1.023	2.046	30.69
GPS L5	1176.45	BPSK (10)	10.23	20.46	24
Galileo E1 OS	1575.42	CBOC (6,1,1/11)	1.023	14.322	24.55
Galileo E5 full	1191.795	AltBOC (15,10)	10.23	51.15	51.15

## Appendix A. Expressions for the Transformation Matrices Used and Analytic Expressions for the CRB in Short Observation Times

Here, we provide more detail about how the Jacobian transformation matrices, G and A, which were described in Section 3.3 and Section 5, are computed. In addition, we show how the CRB expressions for short observation times discussed in Section 4 were obtained.

As discussed in Section 4.1, the FIM J(ξ), which is expressed using Equations (20) and (26), becomes a diagonal matrix when $\Delta \rho >\rho_{chip}$, i.e.:

(A1) $$J\left(\xi \right)=\frac{2N}{\sigma_{w}^{2}}\mathrm{diag}\left\{{\left[\begin{array}{cccccc} R\left(0\right), & u_{0}^{2}R\left(0\right), & -u_{0}^{2}R^{\prime \prime }\left(0\right), & R\left(0\right), & u_{1}^{2}R\left(0\right), & -u_{1}^{2}R^{\prime \prime }\left(0\right) \end{array}\right]}^{T}\right\}.$$

However, we are interested in computing the CRB for the set unknown of parameters **Ψ**, already defined in Equation (29) as:

(A2) $$\Psi ={\left[u_{0},\phi_{0},\rho_{0},\left|\Gamma \right|,\phi_{\Gamma },h\right]}^{T},$$

by transforming J(ξ) into J(Ψ) (which is not diagonal anymore), as described by Equation (33), i.e.:

(A3) $$J\left(\Psi \right)=GJ\left(\xi \right)G^{T}.$$

As discussed in Section 3.3, the Jacobian transformation matrix G for short observation intervals and this specific case with phase coherence between both signal components is expressed as:

(A4) $$G=\left\{\frac{\partial \xi_{j}}{\partial \Psi_{i}}\right\}=\left[\begin{array}{cccccc} 1 & 0 & 0 & \left|\Gamma \right| & 0 & 0\\ 0 & 1 & 0 & 0 & 1 & 0\\ 0 & 0 & c^{-1} & 0 & 0 & c^{-1}\\ 0 & 0 & 0 & u_{0} & 0 & 0\\ 0 & 0 & 0 & 0 & 1 & 0\\ 0 & 0 & 0 & 0 & -2k\sin\left(\theta_{el}\right) & 2\sin\left(\theta_{el}\right)c^{-1} \end{array}\right].$$

With some algebra, the CRB for **Ψ** is then obtained as:

(A5) $$\mathrm{CRB}\left(\Psi \right)={\left[J^{-1}\left(\Psi \right)\right]}_{ii}=\frac{\sigma_{w}^{2}}{2N}\left[\begin{array}{c} \frac{1}{R(0)}\\ \frac{1}{u_{0}^{2}R\left(0\right)}\\ -\frac{c^{2}}{u_{0}^{2}R^{\prime \prime }\left(0\right)}\\ \frac{{\left|\Gamma \right|}^{2}+1}{u_{0}^{2}R\left(0\right)}\\ \frac{\left(R^{\prime \prime }\left(0\right)-R\left(0\right)k^{2}c^{2}\right)\left({\left|\Gamma \right|}^{2}+1\right)}{u_{0}^{2}{\left|\Gamma \right|}^{2}R\left(0\right)R^{\prime \prime }\left(0\right)}\\ \frac{-c^{2}\left({\left|\Gamma \right|}^{2}+1\right)}{4u_{0}^{2}{\left|\Gamma \right|}^{2}\sin^{2}\left(\theta_{el}\right)R^{\prime \prime }\left(0\right)} \end{array}\right].$$

CRB(h), CRB(|Γ|), $\mathrm{CRB}(\phi_{\Gamma })$, defined in Expressions (35), (36) and (39), are obtained in a straightforward manner from the 3rd, 4th and 5th elements of Expression (A5), respectively.

The CRB expression presented in Equation (41) for the phase altimetry case, described in Section 4.1.1, is obtained by removing the fifth row and the fifth column (both related to $\Phi_{\Gamma }$) from J(Ψ) and then computing the inverse of this matrix. Now, the ${\mathrm{CRB}}_{alt}\left(h\right)$ is the element related to *h* in the main diagonal, i.e., the fifth element. In the same way, the CRB for the reflection coefficient estimation case, i.e., ${\mathrm{CRB}}_{r}$, described in Section 4.1.2, with Equations (42) and (43), is obtained following the same procedure, but now removing the last row and the last column (both related to *h*) from J(Ψ).

In Section 5, the Jacobian transformation matrix $A\in R^{6L\times P}$ was introduced, with *L* being the number of $T_{coh}$ intervals considered and *P* the number of parameters in $\Psi_{long}$. For a $s_{\Gamma }={\left[\varepsilon_{r},\sigma \right]}^{T}$, as in Section 6, we have the following:

(A6) $$A=\left\{\frac{\partial \Psi_{ext_{j}}}{\partial \Psi_{long_{i}}}\right\}=\left[\begin{array}{cccccc} \delta_{0} & 0 & \ldots & & & 0\\ 0 & \delta_{0} & 0 & \ldots & & 0\\ \vdots & & & & & \vdots \\ 0 & & & & 0 & \delta_{0}\\ \delta_{1} & 0 & \ldots & & & 0\\ 0 & \delta_{1} & 0 & \ldots & & 0\\ \vdots & & & & & \vdots \\ 0 & & & & 0 & \delta_{1}\\ \delta_{2} & 0 & \ldots & & & 0\\ 0 & \delta_{2} & 0 & \ldots & & 0\\ \vdots & & & & & \vdots \\ 0 & & & & 0 & \delta_{2}\\ \frac{\partial \left|\Gamma \left(\theta_{el}\left[0\right]\right)\right|}{\partial \varepsilon_{r}}\delta_{3} & \frac{\partial \left|\Gamma \left(\theta_{el}\left[1\right]\right)\right|}{\partial \varepsilon_{r}}\delta_{3} & \ldots & & & \frac{\partial \left|\Gamma \left(\theta_{el}\left[L-1\right]\right)\right|}{\partial \varepsilon_{r}}\delta_{3}\\ \frac{\partial \phi_{\Gamma }\left(\theta_{el}\left[0\right]\right)}{\partial \sigma }\delta_{4} & \frac{\partial \phi_{\Gamma }\left(\theta_{el}\left[1\right]\right)}{\partial \sigma }\delta_{4} & \ldots & & & \frac{\partial \phi_{\Gamma }\left(\theta_{el}\left[L-1\right]\right)}{\partial \sigma }\delta_{4}\\ \delta_{5} & \delta_{5} & \ldots & & & \delta_{5} \end{array}\right],$$

where the vector $\delta_{k}\in R^{1\times 6}$ has been defined as a row-vector with all elements equal to zero, except for the position indicated by the subindex k∈{0,…,5}, which has a value of one. $\left|\Gamma (\theta_{el}\left[l\right])\right|$ and $\phi_{\Gamma }\left(\theta_{el}\left[l\right]\right)$ will change together with $\theta_{el}$ in each of the *L* intervals of $T_{coh}$ duration, over the total observation time.

## Appendix B. Zavorotny–Larson Model for the Surface Reflection Coefficient Γ

In [7], the authors proposed a model for the complex reflection coefficient $\Gamma =|\Gamma |\exp\left\{\phi_{\Gamma }\right\}$ of the GPS L1 signal for the case of bare soil with a plane surface. Γ is defined like in Expression (30) as the amplitude ratio between the reflected and the LOS signals. The model treats the soil as a single-layer dielectric medium with losses. It also takes into account the polarization dependent antenna gain effect on the reflected signal. The main geometric optic contribution to the amplitude of the reflected signal comes from the first Fresnel zone around the specular reflection point. According to the model, when the antenna boresight is perpendicular to the ground, Γ can be expressed as a function of the satellite elevation $\theta_{el}$ as:

(B1) $$\begin{array}{cc} \Gamma (\theta_{el}) & =\exp\left\{-0.5\;k^{2}\sigma_{s_{h}}^{2}\sin^{2}\left(\theta_{el}\right)\right\}\\ & \;\times \left(V_{RR}F_{R}\left(-\theta_{el}\right)+V_{RL}F_{L}\left(-\theta_{el}\right)\right), \end{array}$$

where the effect of the surface roughness is incorporated by the exponential term, the so-called coherence loss factor [61]. k=2π/λ is the carrier wavenumber, and $\sigma_{s_{h}}$ is the standard deviation of the surface height. $F_{R}\left(\theta_{el}\right)$ and $F_{L}\left(\theta_{el}\right)$ represent the antenna complex amplitude gains for right-hand and left-hand circular polarizations (RHCP and LHCP) at $\theta_{el}$, respectively; and

(B2) $$\begin{array}{cc} V_{RR} & =V_{LL}=\frac{1}{2}\left(V_{v}+V_{h}\right),\\ V_{RL} & =V_{LR}=\frac{1}{2}\left(V_{v}-V_{h}\right), \end{array}$$

are the polarization-dependent reflection coefficients, where the first subindex stands for the polarization state (*R* for RHCP and *L* for LHCP) of the incident wave, while the second subindex stands for the polarization state of the reflected wave. $V_{v}$ and $V_{h}$ are the coefficient for the linear vertical and horizontal polarizations, which can be expressed as:

(B3) $$\begin{array}{cc} V_{v} & =\frac{\varepsilon \sin\left(\theta_{el}\right)-\sqrt{\varepsilon -\cos^{2}\left(\theta_{el}\right)}}{\varepsilon \sin\left(\theta_{el}\right)+\sqrt{\varepsilon -\cos^{2}\left(\theta_{el}\right)}},\\ V_{h} & =\frac{\sin\left(\theta_{el}\right)-\sqrt{\varepsilon -\cos^{2}\left(\theta_{el}\right)}}{\sin\left(\theta_{el}\right)+\sqrt{\varepsilon -\cos^{2}\left(\theta_{el}\right)}}, \end{array}$$

with:

(B4) $$\varepsilon \approx \varepsilon_{r}-j60\lambda \sigma$$

the complex dielectric permittivity of the material [81,82]. In Equation (B4), $\varepsilon_{r}$ represents the real-part of the permittivity, and *σ* is the conductivity of the reflection surface (in S/m units). The model for Γ can be extended for the multiple layer ground case, where Γ will also depend on the thickness of each layer and their relative permittivity [81,83]. For example in [84,85], the authors use a three-layer (air + two soil layers) model for GNSS-R applications working with the GPS L1 band signal. For more details on all of the assumptions made to obtain (B1), we refer the interested reader to [7].
